# Retraction Note: Mesenchymal stem/stromal cells as a valuable source for the treatment of immune-mediated disorders

**DOI:** 10.1186/s13287-024-03733-0

**Published:** 2024-04-22

**Authors:** Alexander Markov, Lakshmi Thangavelu, Surendar Aravindhan, Angelina Olegovna Zekiy, Mostafa Jarahian, Max Stanley Chartrand, Yashwant Pathak, Faroogh Marofi, Somayeh Shamlou, Ali Hassanzadeh

**Affiliations:** 1 Tyumen State Medical University, Tyumen, Russian Federation; 2 Department of Pharmacology, Saveetha Dental College and Hospital, Saveetha Institute of Medical and Technical Sciences, Saveetha University, Chennai, India; 3 Department of Pharmacology, Saveetha Dental College and Hospital, Saveetha Institute of Medical and Technical Sciences, Chennai, India; 4 Department of Prosthetic Dentistry, Sechenov First Moscow State Medical University, Moscow, Russia; 5 German Cancer Research Center, Toxicology and Chemotherapy Unit (G401), 69120 Heidelberg, Germany; 6 DigiCare Behavioral Research, Casa Grande, AZ USA; 7 Professor and Associate Dean for Faculty Affairs, Taneja College of Pharmacy, University of South Florida, Tampa, FL USA; 8 Department of Hematology, Faculty of Medicine, Tabriz University of Medical Sciences, Tabriz, Iran; 9 Department of Applied Cell Sciences, School of Advanced Technologies in Medicine, Tehran University of Medical Sciences, Tehran, Iran; 10 Cell Therapy and Regenerative Medicine Research Center, Tehran University of Medical Sciences, Tehran, Iran; 11 Department of Tissue Engineering and Applied Cell Sciences, School of Advanced Technologies in Medicine, Tehran University of Medical Sciences, Tehran, Iran

**Retraction Note: Stem Cell Research & Therapy (2021) 12:192**

10.1186/s13287-021-02265-1

The Editors-in-Chief have retracted this article. An investigation by the Publisher has found a number of articles, including this one, which share similar concerns, involving but not limited to, irregularities with respect to submission and authorship. The Editors-in-Chief therefore no longer have confidence in the results and conclusions presented in this article. Alexander Markov, Lakshmi Thangavelu, and Ali Hassanzadeh do not agree to this retraction. Surendar Aravindhan, Angelina Olegovna Zekiy, Mostafa Jarahian, Max Stanley Chartrand, Yashwant Pathak, Faroogh Marofi and Somayeh Shamlou did not respond to correspondence from the Editor about this retraction.

